# A Comparative Study Evaluating the Effectiveness Between Ketogenic and Low-Carbohydrate Diets on Glycemic and Weight Control in Patients With Type 2 Diabetes Mellitus: A Systematic Review and Meta-Analysis

**DOI:** 10.7759/cureus.25528

**Published:** 2022-05-31

**Authors:** Hany A Zaki, Haris Iftikhar, Khalid Bashir, Hesham Gad, Ahmed Samir Fahmy, Amr Elmoheen

**Affiliations:** 1 Emergency Medicine, Hamad Medical Corporation, Doha, QAT; 2 Medicine, Qatar University, Doha, QAT; 3 Endocrinology and Diabetes, Arab Contractors Medical Center, Cairo, EGY; 4 Nutrition, Egyptian Ministry of Health, Tanta, EGY

**Keywords:** systematic review and meta analysis, weight control, glycemic level, diabetes mellitus type 2, low-carb diets, ketogenic diet

## Abstract

Diabetes mellitus (DM) has become a worldwide public health burden and a significant cause of motility and morbidity. The most common type of diabetes is type 2 diabetes, which is estimated to have a prevalence of one in every ten adults living with diabetes in the United States. The risk factors for type 2 diabetes are obesity and being overweight. Therefore, the primary strategy used to manage type 2 diabetes is weight loss. Different measures, such as dietary therapies and physical training, have been used to manage type 2 diabetes through weight and glycemic control. The dietary therapies used to manage type 2 diabetes are ketogenic and low-carbohydrate diets. Despite studies showing that both ketogenic and low-carbohydrate diets significantly impact weight and glycemic control, the difference between the two diets has not been fully established. Therefore, this systematic review has demonstrated and compared the effectiveness of ketogenic and low-carbohydrate diets on glycemic and weight control.

The literature search was conducted on five electronic databases, PubMed, ScienceDirect, Embase, Web of Science, and Google Scholar, from 2000 to 2022. Specified keywords related to the ketogenic diet (KD), low carbohydrates, and type 2 diabetes were used to search for relevant and original articles. The identified articles were analyzed using the eligibility criteria before they were included in the study. The eligibility criteria yielded 15 studies that were included in this systematic review. The results obtained by conducting a meta-analysis showed that low-carbohydrates had a greater reduction in the HbA1c than other diets (standardized mean difference [SMD]: -0.27%; 95% CI; -0.60%, 0.07%: P = 0.008, I2 = 66%). Similarly, a significant decrease in HbA1c percentage was recorded in patients that consumed KDs compared to those who consumed the control diets (SMD: -1.45%; 95% CI; -2.73%, -0.17%: P < 0.00001). The results also show that the KD significantly impacts weight loss than control diets.

The results show that the KD is more effective in reducing glycated haemoglobin and body weight (BW) than a low-carbohydrate diet. Therefore, we can summarize that the KD is a more effective dietary therapy. However, there is a need to balance the weight loss and glycemic control benefits obtained from the KD with the increased cardiovascular risks for patients with type 2 diabetes.

## Introduction and background

Diabetes mellitus (DM) has become a worldwide public health burden and a major cause of motility and morbidity. Data from the International Diabetes Federation (IDF) Diabetes Atlas 10th edition shows that in 2021, about 6.7 million people died from diabetes, which is one person every five seconds. As of 2021, the prevalence of DM was estimated to be over 537 million adults (aged 20-79) globally. The prevalence is expected to increase to 643 million by 2030 and 783 million by 2045 [1]. In the United States, type 2 diabetes mellitus (T2DM) is rapidly growing and is estimated to have a prevalence of about one in every ten adults living with diabetes [2]. The main risk factors associated with T2DM are being overweight and obesity, which is present in about 80% of patients living with T2DM. The primary strategy that can be used to manage T2DM is weight loss, which can be achieved using dietary therapy, preferably combined with physical training. Studies report that the benefits of weight loss in managing T2DM are not limited to glycemic control but also other cardiovascular risk factors such as blood pressure, high-density lipoprotein (HDL), total cholesterol (TC), and triglyceride (TG) [3]. Patients with T2DM have also been found to be insulin resistant, which can be reduced by weight loss.

Physicians have implemented numerous dietary therapies, including low carbohydrate and ketogenic diets (KD), to manage T2DM. KD has gained interest as a quick and effective weight loss strategy. In 1920, KD was initially proposed as a treatment option for drug-resistant epilepsy among young patients, which to date is still the only validated clinical indication [4]. KD is defined as a very low carbohydrate (<50 g/day or <10 g/day energy intake), high-fat (>75% of total energy) diet which induces nutritional ketosis among patients. This induced ketosis has an appetite-suppressing effect; therefore, many patients have been prompted to use this diet to aid in weight loss [5]. Additionally, among patients with diabetes, KD has been used due to its effect on blood glucose and weight. Several studies have reported that patients who have followed ketogenic dietary therapy have positive health outcomes such as weight loss, improved glycemic control, and decreased medication dosages [6,7]. A previous systematic review of nine randomized control trials (RCTs) reported that very low-carbohydrate diets significantly improved short-term weight and reduced glycated haemoglobin compared to high or normal carbohydrate diets [8]. The study also reports that overweight patients with T2DM had a significant reduction or cessation of diabetes-related medications.

Recently, low-carbohydrate diets have also gained popularity because of their effect on weight loss, improvement of glycated haemoglobin (HBA1c), and reduction or cessation of medication. A previous systematic review and meta-analysis of randomized trials that compared low-carbohydrate and high-carbohydrate diets showed that low-carbohydrate diets significantly improved HBA1c among adult patients with T2DM compared to high-carbohydrate diets [9]. Despite studies showing that both KD and low-carbohydrate diets significantly impact weight and glycemic control, the difference between the two diets has not been fully established. Therefore, this systematic review and meta-analysis will address and compare the effectiveness of low-carbohydrate and KDs among patients with T2DM. This systematic review hypothesizes that ketogenic and low-carbohydrate diets will improve glycemic measures, i.e., HBA1c, and decrease BMI and body weight among patients with T2DM.

## Review

Methods

Literature Search and Reporting

For relevant randomised clinal trials and other primary studies, searches were conducted on five electronic databases, PubMed, ScienceDirect, Embase, Web of Science, and Google Scholar. In addition, the reference lists of identified studies were also scoured for additional studies. All the searches were conducted per Preferred Reporting Items for Systematic Reviews and Meta-analyses (PRISMA) guidelines and priori protocol from the International Prospective Register of Systematic Reviews (PROSPERO). Key search terms and the Boolean operators “AND” and “OR” were used for an effective literature search. The search strategy was as follows; (low carbohydrate OR low-carb) AND (ketogenic OR very low carbohydrate) AND (glycemic OR glucose OR HBA1c) AND (weight OR BMI OR body mass index) AND (type 2 diabetes OR T2DM). The search query was conducted from 2000 to 2022.

Eligibility Criteria

Two reviewers were tasked with screening all the articles based on the inclusion and exclusion criteria. For studies to be included in this review, they had to meet the following inclusion criteria: studies written and published in English, studies that compared either low-carb diets or KDs to other diets in patients with type 2 diabetes, studies conducted only on human beings, and studies with ten or more patients. The exclusion criteria were outlined below: studies conducted on animal species and studies published in other languages. This consideration was made to avoid translation of scientific works which could lose meaning or context, studies that compared other dietary therapies other than low-carb and KDs, and studies that evaluated the dietary therapies on patients other than type 2 diabetes patients (e.g., type 1 diabetes patients, obesity, etc.)

Data Extraction

Two reviewers independently retrieved and compiled all the relevant data from the included studies. Data retrieved from these studies included author ID (names and year of publishment), study type, population (sample size, age, and sex), intervention, control intervention, and main results. The main outcomes for this systematic review and meta-analysis included HBA1c used as a measure for glycemic control and BMI and body weight (BW) used as measures for weight control. Any discrepancies in the data extraction process were reconciled by consulting a third reviewer.

Quality Assessment

**Figure 1:**
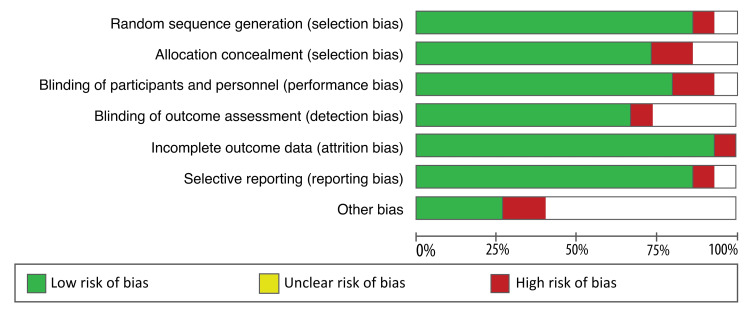
Risk of bias graph.

**Figure 2:**
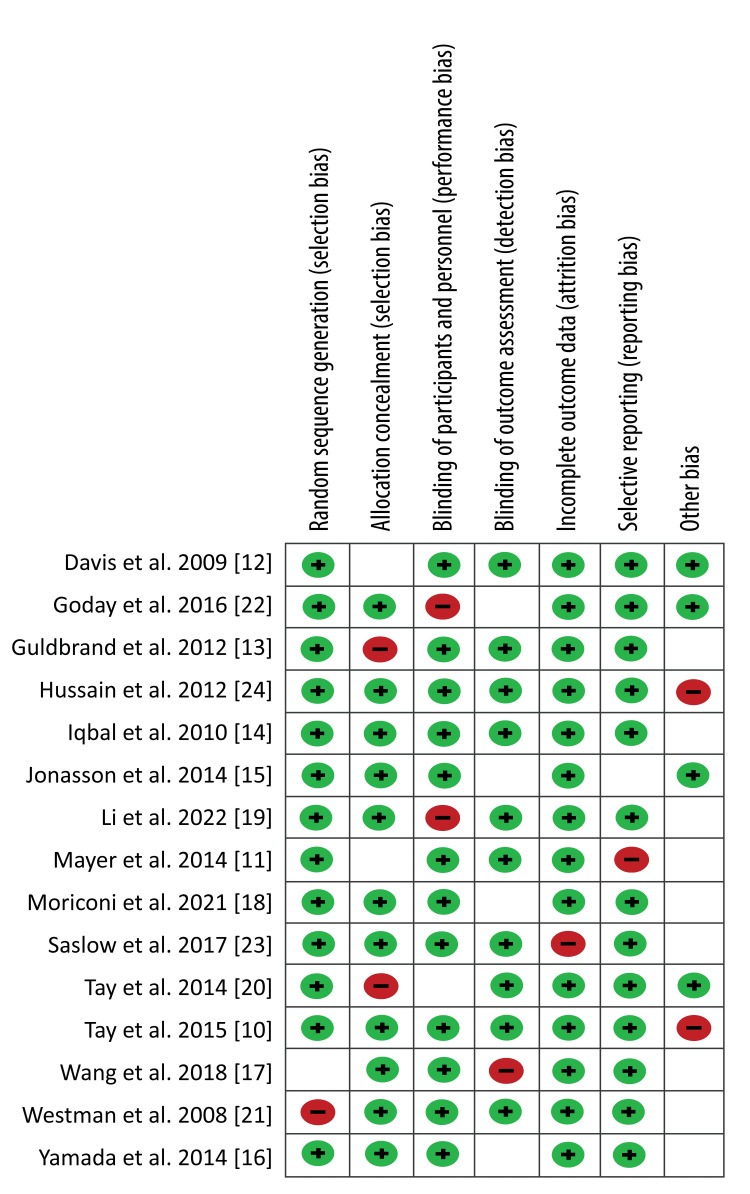
Risk of bias summary.

A quality assessment was conducted on all included studies based on the specific criteria outlined in the Cochrane Handbook for Systematic Reviews of Interventions. A ReviewManager (RevMan 5.4.1, Cochrane, Alberta, Canada) software was utilised to accomplish a quality assessment of included studies. The quality of each study was categorised into; “low risk,” “high risk,” and “unclear risk” by considering the elements of selection, performance, attrition, and reporting bias. A study with sufficient and relevant results was considered “good” and was said to have a low risk of bias. Conversely, a study with insufficient reporting was reported to have a high risk of bias. On the other hand, a lack of clear judgment from the reviewers resulted in considering a study to be of unclear risk. Below is Figure 1, which shows the risk of bias graph and Figure 2, which shows the risk of bias summary; both figures summarise the quality assessment of articles included in this review. 

Data Analysis

ReviewManager (RevMan 5.4.1) software was used to conduct a meta-analysis on all the outcome measures. The effect size of BMI, BW loss, and HBA1c was calculated using standardised mean difference (SMD). The 95% CI for each SMD was also calculated. The study population was used as the weight for all data calculations. The I2 statistics were used in the calculation of data heterogeneity. Forest plots were used to show the statistical analysis of all the outcome measures.

Results

Study Selection

**Figure 3:**
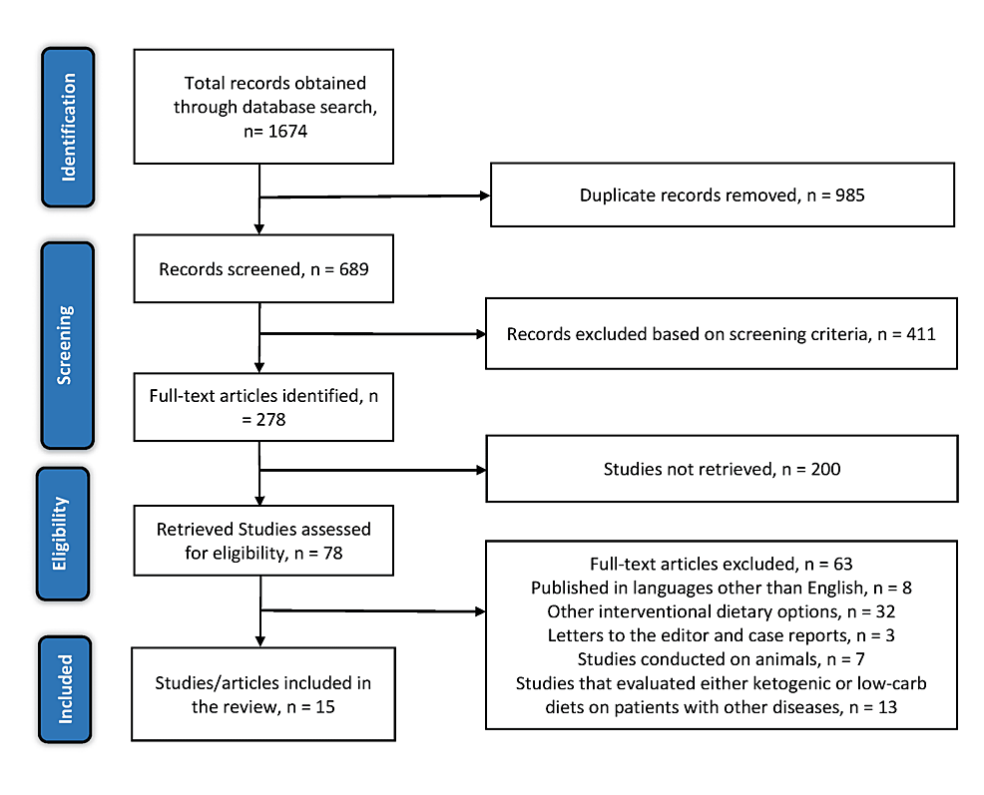
PRISMA flow diagram of the literature search results. PRISMA: Preferred Reporting Items for Systematic Reviews and Meta-Analysis.

The initial electronic database search yielded 1674 articles. Of the 1674 articles, 985 were excluded since they were duplicates. The titles and abstracts of the remaining 689 articles were screened, of which only 278 articles met the screening criteria. Two hundred articles were not retrieved. The remaining 78 articles were assessed using the eligibility criteria, and only 16 articles met the criteria. Of the 78 articles, ten were excluded since they were published in other languages, six were conducted on animals, and nine evaluated either ketogenic or low-carbohydrate diets on other patients instead of patients with type 2 diabetes. Eight evaluated other interventional diets other than ketogenic or low-carbohydrate diets, 13 were systematic reviews, letters to the editor, or case reports, and six evaluated less than ten patients. Figure 3 shows a PRISMA flow diagram, and Table 1 shows the study characteristics. 

**Table 1:** The study characteristics. RCT: Randomized control trial; LC: Low carbohydrate; HC: High carbohydrate; T2DM: Type 2 diabetes mellitus; LFD: Low-fat diet; HbA1c: Hemoglobin A1c Test; VLCKD: Very-low-calorie ketogenic diet.

Author ID	Study Design	Participants	Interventional Diet	Comparison Diet	Main Outcomes
Tay J et al. [10]	RCT	78 obese patients (age 35-68 years) with type 2 diabetes mellitus (T2DM) completed the trial and were studied. 41 patients were in the low-carbohydrate group while 37 were in the high-carbohydrate group.	Low carbohydrate (LC) (14% of total energy from carbohydrate, 28% protein and 58% fat (35% monounsaturated fat and 135 polyunsaturated fat))	High carbohydrate (HC) (53% carbohydrate, 17% protein, and <30% fat (15% monounsaturated fat and 9% polyunsaturated fat))	LC and HC diets significantly reduced the HbA1c (-1.0 (-1.2, -0.7) and -1.0 (-1.3, -0.8), respectively) and fasting blood glucose (-0.7 (-1.3, -0.1) -1.5 (-2.1, -0.8), respectively). An overall weight loss of 9.1% was observed in both groups.
Mayer SB et al. [11]	RCT	46 patients with T2DM took part in the trial. 22 were assigned to the low-carbohydrate group, while 24 were assigned low-fat diet plus orlistat (LFD + O)	LC (<=20g carbohydrate intake daily)	LFD + O (<30% of total fat energy intake, <10% saturated fat, <300mg cholesterol, 500-100okcal deficit and 120 mg orlistat taken 3 times daily)	BMI and weight were significantly and similarly reduced in LC and LFD + O groups, i.e., BMI (36.3 and 37.3 kg/m2 in LC and LFD + O groups, respectively) and weight (109.4 and 117.0 kg, respectively). A significant decrease in HbA1c was observed in the LC group (from 7.6% to 6.9%) compared to LFD + O group (from 7.6% to 7.7%). Fasting glucose significantly reduced in LC group (from 152.6 to 133.7 mg/dl) compared to LFD + O group (from 149.0 to 146.8 mg/dl).
Davis NJ et al. [12]	RCT	105 obese patients with T2DM were studied. LC group had 55 participants, while LFD had 50 patients.	LC (20-25g carbohydrate intake daily for a 2-week phase and 5g increment in carbohydrate intake depending on weight loss)	LFD (25% of energy from fat intake depending on baseline weight).	An overall decrease in A1c of 0.12% per month was observed in both groups in the early phase (0-3 months); however, during the late phase (3-12 months), an average increase of 0.06 per month was observed. During the earlier phase, an average weight loss of 1.7kg/month was observed in the LC group; however, the patients gained 1.2kg/month during the late phase. A slow weight loss of 1.2kg/month was observed in the LFD group during the early phase and the weight loss plateaued during the late phase with an average weight gain of <0.01 kg/month.
Guldbrand H et al. [13]	RCT	61 patients with T2DM took part in the study. 31 patients were allocated to the LFD group, while 30 patients were allocated to the LC group.	LC (20% energy from carbohydrates, 50% from fat, and 30% from proteins)	LFD (30% of energy from fat (<10% energy from saturated fat), 55-60% from carbohydrates, and 10-15% from protein.)	Patients in the LFD group were observed to have a weight loss of 3.1±4.3 kg compared to a weight loss of 3.5±4.0 kg observed in the LC group after 24 months of dietary intervention. The BMI values showed no difference statistically in the LFD and LC groups (30.5±5 and 29.8±4.5, respectively). No statistical difference in HbA1c was observed in LFD and LC groups after 24 months (7.5±3.1 and 7.5±2.9, respectively).
Iqbal N et al. [14]	RCT	144 T2DM patients (mean age of 59.4 years) participated in the trial. 70 participants were allocated to the LC group, while 74 were allocated to LFD.	LC (30g carbohydrate intake daily)	LFD (<7% of total calories from saturated fats and <300mg cholesterol consumption daily)	After 6,12, and 24 months, no statistical difference was observed between the groups; however, at 24 months, participants in the LC and LFD groups lost 1.5 and 0.2 kg, respectively. A significant decrease of -0.5% in HbA1c was observed among patients in the LC group compared to a -0.1% decrease observed in the LFD group.
Jonasson L et al. [15]	RCT	61 patients with T2DM were studied. 31 patients (13 males and 18 females) were allocated to the LFD group, while 30 patients (14 males and 16 females) were allocated to the LC group	LC (20% of energy from carbohydrates)	LFD (30% of energy from fat)	A significant and similar difference in BMI was observed in LFD (from 34 to 32 kg/m^2^) and LC (from 32 to 30 kg/m^2^) groups. A reduction in HbA1c was observed in LC and LFD groups; however, the difference was not statistically significant (56 and 57 mmol/l, respectively).
Yamada Y et al. [16]	RCT	24 patients (mean age, 63.3±11.7 years) with T2DM were recruited for the study. The LC group had 12 patients (7 male and 5 female), while the calorie-restricted diet group had 12 patients (5 males and 7 female)	LC (<130g daily intake of carbohydrates)	Calorie restricted diet	The change in body weight and BMI were not significant in either group, i.e., BMI changed from 24.5 ± 4.3 to 23.6 ± 3.5 mg/dL for patients in the LC group and from 27.0 ± 3.0 to 26.4 ± 2.2 mg/dL for patients in the calorie-restricted diet group. Body weight for patients in the LC group changed from 67.0 ± 15.9 to 64.4 ± 14.2 kg, while for patients on a calorie-restricted diet, the body weight changed from 68.1 ± 7.7 to 66.7 ± 7.0 kg. LC group recorded a significant decrease in HbA1c levels (from 7.6 ± 0.4% to 7.0 ± 0.7%) compared to calorie restricted group (from 7.7 ± 0.6% to s 7.5 ± 1.0%).
Wang LL et al. [17]	Prospective RCT	56 patients with T2DM were recruited for the study. The LC and LFD groups had 28 patients each.	Low carbohydrate diet	Low-fat diet	HbA1c levels significantly reduced was observed in the LC and LFD group (0.63 ± 1.18% and 0.31 ± 0.70%, respectively). The fasting glucose level was improved significantly in both groups; however, the difference was statistically insignificant (6.87 ± 0.65 and 6.70 ± 0.57, for LC and LFD groups, respectively).
Moriconi E et al. [18]	Retrospective observational study.	30 obese participants (age 35-75 years) with T2DM participated. 15 patients (7 female and 8 men) were in the very-low-calorie ketogenic diet (VLCKD) group, and 15 patients (7 female and 8 male) were in the low-calorie diet (LCD) group.	VLCKD (During the first phase, patients were required to have a total energy intake of <800kcal and a daily protein intake of between 1.2 and 1.5 kg. during the second phase, conventional food consisting of proteins was introduced )	LCD (a daily reduction of 500-1000 kcal in energy intake. 30% calories from fat (<7% kcal/day of saturated fat, 10-20% kcal/day of polyunsaturated fatty acids, 10-20% monounsaturated fatty acids, and <300 mg/day of cholesterol), 20– 25% from protein and 45–50% from carbohydrates.	After 3 (T1) and 12 (T2) months, a significant weight loss of 3kg was observed in patients in the VLCKD group, while patients in the LCD group showed no significant change. A decrease of 0.69 ± 0.65% in HbA1c was observed among patients in the VLCKD group, while a decrease of 0.42 ± 0.01% was observed in the LCD group after 3 months. This difference was statistically insignificant.
Li S et al. [19]	RCT	The study involved 53 patients (aged 18-50 years) newly diagnosed with T2DM. The ketogenic diet (KD) group had 24 patients, while the diabetic diet group had 29 patients	KD (daily intake; 30-50g of carbohydrate, 60g protein, 130g fat, and 1500±50 kcal of total calories)	Diabetic diet (daily intake; 250-280g carbohydrate, 60g protein, 20g fat, and 1500±50 kcal of total calories)	A decrease in BMI and HbA1c was observed in both groups after the intervention; however, the difference was not statistically significant, i.e., HbA1c decreased from 8.74±1.63% to 7.82±1.43% in the KD group while the decrease in diabetic diet group was from 8.69±1.59% to 8.42±1.51%. BMI decreased from 29.04±5.81 to 26.21±5.74 kg/m^2^ in the KD group, while for patients in the control group BMI decreased from 29.75±6.07 to 29.42±5.97 kg/m^2^.
Tay J et al. [20]	RCT	The primary analysis was conducted on 93 obese/overweight patients with T2DM. 46 patients were in the very low-carbohydrate diet group (ketogenic diet), while 47 patients were in the high-carbohydrate group.	Very-low-carbohydrate diet (14% total energy from carbohydrates, 28% protein, and 58% total fat (35% monounsaturated fat and 13% polyunsaturated fat)	HC (53% total energy from carbohydrates, 17% protein, and <30% total fat (15% monounsaturated fat and 9% polyunsaturated fat)	A significant difference in the BMI values was observed in the very-low-carbohydrate diet and high-carbohydrate group (-4.0 (2.0) and -4.0 (1.8), respectively); however, the difference between the two groups is insignificant. HbA1c levels were reduced to a greater extent in the very-low-carbohydrate diet group for patients with baseline HbA1c > 7.8%. The decrease in fasting glucose was insignificant in the two groups (-1.1 (2.2) and -1.6 (2.5) for LC and HC diets, respectively).
Westman EC et al. [21]	RCT	50 participants with T2DM completed the trial. 29 patients were in the low-glycaemic, reduced-calorie diet (LGID), while 21 patients were in the low-carbohydrate, ketogenic diet (LCKD)	LCKD (< 20g daily intake of dietary carbohydrate)	LGID (~55% daily intake of carbohydrates)	A greater decrease in HbA1c levels from baseline was observed for patients in the LCKD group (8.8 ± 1.8% to 7.3 ± 1.5%) compared to patients in the LGID group (8.3 ± 1.9% to 7.8 ± 2.1%). A significantly greater weight loss was observed in the LCKD group (from 108.4 ± 20.5 kg to 97.3 ± 17.6 kg) compared to the LGID group (from 105.2 ± 19.8 to 98.3 ± 20.3 kg).
Goday A et al. [22]	RCT	89 patients (aged 30-60 years; 31 men and 58 women) with T2DM were studied. 45 patients were allocated to the very low-carbohydrate ketogenic (VLCK) diet, while 44 were allocated to the low-calorie diet.	VLCK (a very low-calorie diet consisting of 600-800 kcal/day, low carbohydrate of <50g/day from vegetables and lipids (10g from olive oil daily).	Low-calorie diet (calorie restriction of 500-1000 kcal/day)	A greater body weight loss was observed among patients in the VLCK group (from 91.5 (11.4) to 76.8 (9.1) kg) compared to patients in the low-calorie diet group (from 90.0 (11.3) to 84.95 (13.6) kg). The HbA1c was significantly decreased in patients in the VLCK group (from 6.9 (1.1) 6.0 (0.7) %) compared to patients in the low-calorie diet group (from 6.8 (1.0) to 6.4 (0.8) %).
Saslow LR et al. [23]	RCT	29 of 34 patients (age >= 18 years) with T2DM completed the trial and were studied. The low-carbohydrate ketogenic diet (LCK) had 14 patients, while the moderate-carbohydrate, calorie-restricted, low-fat diet group (MCCR) had 15 patients.	LCK (restriction of 20-50g of carbohydrate intake daily)	MCCR (45-50% of total calories from carbohydrates)	After 12 months of intervention, patients in the LCK group had an 8.3% body weight loss, while patients in the MCCR had a 3.8% body weight loss. A greater decrease in HbA1c levels was observed in patients in the LCK group (from 6.6 (6.3, 6.9) to 6.1 (5.8, 6.4) %) compared to patients in MCCR group (from 6.9 (6.6, 7.2) to 6.7 (6.4, 7.0) %).
Hussain TA et al. [24]	RCT	The study was conducted on 363 patients, of which 102 were diagnosed with T2DM. The low-calorie diet group had 24 T2DM patients, low-carbohydrate ketogenic diet group (LCKD) had 78.	LCKD (patients were restricted to ~20g of carbohydrate intake daily)	Low-calorie diet	A significant body weight loss was observed among diabetic patients in the LCKD group (from 104.01 ± 18.89 to 91.56 ± 17.45 kg) compared to patients in the low-calorie diet group (from 95.71 ± 9.56 to 89.02 ± 5.97 kg). There was no significant difference in BMI values observed among diabetic patients in the LCKD group (from 36.31 ± 2.63 to 33.87 ± 2.75 kg/m^2^) compared to diabetic patients in the low-calorie diet group (from 39.84 ± 6.40 to 35.05 ± 5.90 kg/m^2^).

Glycemic Control

**Figure 4:**
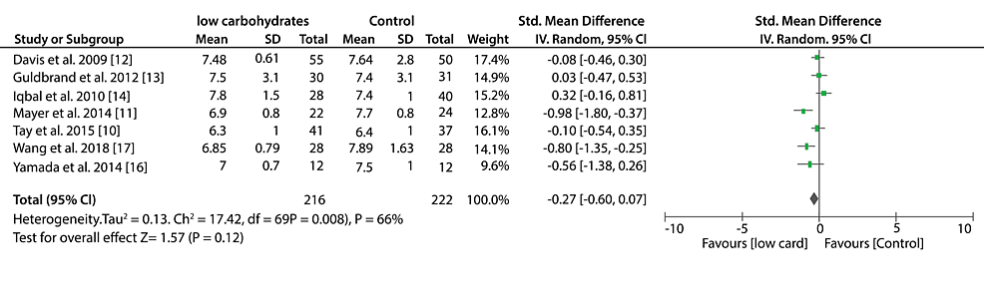
Forest plot showing the effect of a low-carbohydrate diet on HbA1c.

**Figure 5:**
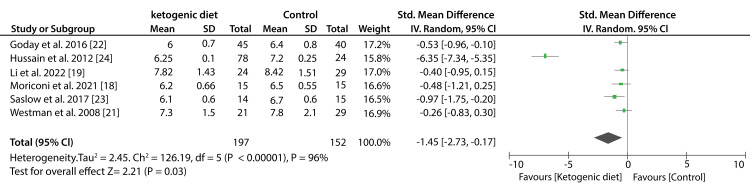
Forest plot showing the effect of ketogenic diets on HbA1c.

Seven included studies compared the efficacy of low-carbohydrates and other diets on the percentage of HbA1c. The pooled results from these studies show that low-carbohydrates had a greater reduction in the percentage of HbA1c compared to other diets (SMD: -0.27%; 95% CI; -0.60%, 0.07%: P = 0.008, I2 = 66%). On the other hand, data from six studies compared the effect of the KD to other diets on the percentage of HbA1c. A significant decrease in HbA1c percentage was recorded in patients that consumed KDs compared to those who consumed the control diets (SMD: -1.45%; 95% CI; -2.73%, -0.17%: P < 0.00001, I2 = 96%). Figure 4 shows a forest plot (effect of low-carbohydrate diets on HbA1c) and Figure 5 shows a forest plot (effect of KDs on HbA1c).

Weight Control

**Figure 6:**
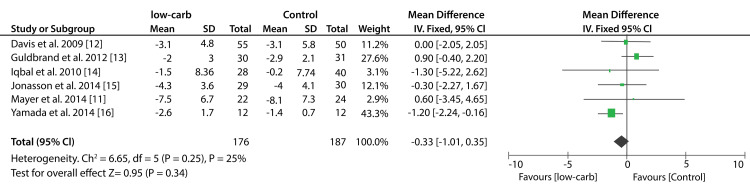
Forest plot showing the effect of a low-carbohydrate diet on body weight.

**Figure 7:**
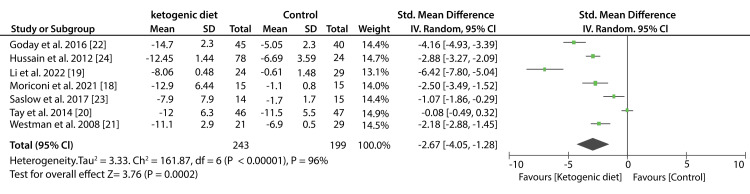
Forest plot showing the effect of ketogenic diet on body weight.

Data from six studies shows that low-carbohydrate diet decreased the BW, however; the difference observed in weight loss was insignificant compared to other diets (SMD: -0.33kg; 95% CI; -1.01kg, 0.35kg; P = 0.25, I2 = 25%). Pooled data from seven studies showed that patients who consumed KDs had a significant weight loss compared to patients who consumed other diets (SMD: -2.67kg; 95% CI; -4.05kg, -1.28kg; P < 0.00001, I2 = 96%). Data from six studies that compared low-carbohydrate to other diets showed that the difference in BMI values was statistically insignificant (SMD; -0.26kg/m2; 95% CI; -0.54kg/m2, 0.01kg/m2; P = 0.20, I2 = 31%). Similarly, data from seven studies showed no significant difference between KDs compared to other diets in the reduction of BMI values (SMD: -0.31kg/m2; 95% CI; -0.81kg/m2, 0.20kg/m2; P < 0.00001, I2 = 84%). Figure 6 shows a forest plot of the effect of a low-carbohydrate diet on BW and Figure 7 shows a forest plot of the effect of a KD on BW.

**Figure 8:**
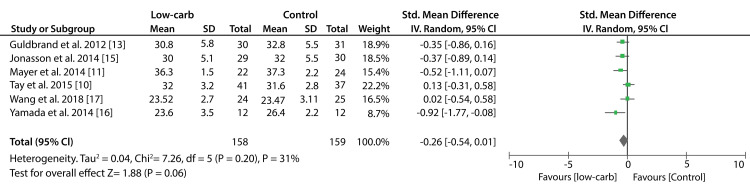
Forest plot showing the effect of a low-carbohydrate diet on BMI.

**Figure 9:**
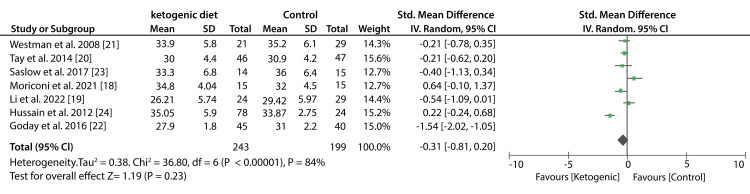
Forest plot showing the effect of a ketogenic diet on BMI.

Figure 8 shows forest plot of the effect of a low-carbohydrate diet on BMI. And lastly Figure 9 shows a forest plot of the effect of a KD on BMI.

Discussion

In this systematic review and meta-analysis, we examined whether low-carbohydrate diets and KDs were effective in weight and glycemic control among patients with type 2 diabetes. Studies evaluated in this review have shown that low-carbohydrate and KDs significantly reduced glycated haemoglobin compared to other control diets. Similarly, KDs showed significant weight loss compared to other diets. However, when compared with other diets, low-carbohydrate diets showed that the difference was statistically insignificant. Ketogenic and low-carbohydrate diets also showed no significant difference in the reduction of BMI values. KDs induce metabolic starvation in the body by limiting the intake of carbohydrates and increasing the consumption of fat [25]. This systematic review and meta-analysis showed that KDs would significantly reduce weight loss and BMI values in T2DM. The pooled results of our meta-analysis have shown that KDs, compared to other dietary therapies, have a significant weight loss of 2.67 kg in patients with T2DM. Similarly, previous reviews have shown significant weight loss among patients with T2DM. For example, a meta-analysis of seven RCTs showed that diabetic patients had a significant weight loss of 7.78 kg after consuming KDs [26]. Similarly, a review of four RCTs conducted by Alarim RA et al. [27] reported that a KD had significant weight loss in patients with T2DM compared to those who were subjected to other diets (SMD: -4.26kg; 95% CI; -6.88kg, -1.63kg; p = 0.001; I2 = 81%). The results in the included studies of this systematic review have shown some inconsistencies. Some studies have reported that KDs have a significant difference in weight loss. For example, a study conducted by Saslow LR et al. [23] reported that after 12 months of intervention, a significant weight loss was observed in the groups that consumed low-carbohydrate ketos and moderate-carbohydrate, calorie-restricted (MCCR), low-fat diet (LFD) (8.3% and 3.8% weight loss, respectively). This shows that the KD had a statistically significant weight loss than the MCCR diet. Other studies have shown no difference between KDs and control diets. Tay J et al. [20] reported that after 24 weeks of consumption of a very low-carbohydrate, low-saturated fat diet, and high-carbohydrate diet, no statistical difference in weight loss was observed in the T2DM patients in the two groups (88.1 (13.7) vs 89.9 (14.9) kg). The pooled results from our meta-analysis have shown that no significant difference in BMI values was observed between KDs and control diets (SMD: -0.31 kg/m2; 95% CI; -0.81 kg/m2, 0.20 kg/m2; P < 0.00001, I2 = 84%). These results are consistent with those of a previous meta-analysis that reported KD reduced BMI values; however, the difference was not statistical (SMD: -0.63kg/m2; 95% CI; -1.31kg/m2, 0.08kg/m2; P < 0.00001, I2 = 87%) [26]. However, results from the included studies showed some variations. Li S et al. [19] reported that after 12-week consumption of KDs, a significant difference in BMI values was observed compared with those subjected to a diabetic diet (26.21 ± 5.74 vs 29.42 ± 5.97, respectively). This significant difference in the BMI was attributed to the fact that the KD stimulated different degrees of starvation and the ketone body became a vital way for energy supply throughout the body.

The pooled results of the meta-analysis affirm our hypothesis that a KD significantly improves HbA1c, which is a glycemic measure. The results show that the HbA1c percentage was reduced by 1.45% after KD consumption. This increased reduction in the HbA1c levels can be attributed to the increased weight loss observed after the consumption of KDs. The results of our meta-analysis are supported by a previous meta-analysis of four RCTs that reported a significant reduction of 1.02% in HbA1c observed in patients that consumed KDs compared to those subjected to other diets [27]. Despite HbA1c levels being used as the measure for glycemic control, fasting glucose is also an important measure to assess the effect of a KD on glycemic control. A randomised study of 53 T2DM patients reported that a significant reduction in fasting glucose was observed in patients subjected to a KD (from 9.01±2.77 to 7.62±1.69 mmol/L) compared to those subjected to a diabetic diet (from 8.98±2.48 to 8.42±2.17 mmol/L). These results are in accordance with the results from a previous study conducted by Myette-Côté É et al. They reported that a KD significantly and rapidly improved the patients’ blood glucose control by lowering the fasting insulin, stabilising blood glucose, and alleviating the fluctuations in blood glucose among patients with T2DM [28]. Similarly, 45 patients with T2DM who consumed very-low-calorie KD showed a significant decrease in fasting glucose (from 136.9 (34.4) to 108.9 (20.4) mg/dL) compared to those who consumed a low-calorie diet (LCD) (from 140.5 (43.1) to 123.3 (24.3) mg/dL) [22].

On the other hand, the results of this systematic review have shown that patients that consumed low-carbohydrate diets had a weight loss of 0.33 kg; however, when compared with the control diets, this difference was not statistically significant. These results are similar to the review conducted by Kirk et al. [29], who reported no significant difference in weight loss after consuming low-carbohydrate diets compared with other diets. Similarly, another meta-analysis reported that the difference in weight loss between low-carbohydrates and control diets was not statistically significant (weighted mean difference (WMD): 20.69 kg; 95% CI: 21.77, 0.39 kg; P = 0.21) [7]. A previous systematic review and meta-analysis of seven RCTs reported that low-to-moderate carbohydrates and high-carbohydrate diets had the same effect on BW after short- and long-term intervention [29]. Some of the included studies have reported that low-carbohydrate diets significantly impact weight loss compared to other diets. A randomised study conducted on 22 patients reported that after a six-month intervention, patients subjected to a low-carbohydrate diet had a significant decrease in BW (from 67.0 ± 15.9 to 64.4 ± 14.2 kg) compared to those subjected to a calorie-restricted diet (from 68.1 ± 7.7 to 66.7 ± 7.0). Other studies have shown that low-carbohydrate diets had an inferior weight loss compared to other diets. For example, a study by Guldbrand H et al. reported that after 12 months of intervention, LFD had a more significant weight loss (from 98.8 ± 21 to 95.9 ± 21 kg) compared to LCD (from 91.4 ± 19 to 89.4 ± 22). However, the study also reports that the short-term (six months) weight loss was not statistically different in either group (-4.0 ± 4.1 kg and -4.3 ± 3.6 kg, for LFD and LCD groups, respectively). No significant difference was observed in BMI values when a low-carbohydrate diet was compared to other diets. The results show that a low-carbohydrate diet lowered BMI by 0.26 kg/m2, which can be considered statistically significant. These results are supported by a previous systematic review that reported a statistical difference in BMI values for patients subjected to a low-carbohydrate diet and a high-carbohydrate diet [29]. The low-carbohydrate diet also reduced HbA1c by 0.27%; however, this difference can be considered not statistically significant. Similarly, results reported in a previous systematic review by Sainsbury E et al. [9] showed that low carbohydrates significantly reduced HbA1c levels compared to patients subjected to high carbohydrates (WMD: -0.36%, 95% CI: -0.62, -0.09). Similarly, a previous study reported a significantly greater reduction in HbA1c for patients in the low-carbohydrate group than in the calorie-restricted diet (-0.65% vs 0.0%) [30]. Another systematic review reported that after six months, a greater reduction in HbA1c levels was observed in diabetic patients receiving a low-carbohydrate diet compared with those in the healthy eating group (-0.4% vs -0.2%) [31].

Limitations of the study

The primary limitation of this systematic review and meta-analysis is that high heterogeneity was observed, especially in studies comparing KDs to other control diets. In some cases, the extent of heterogeneity grew to 96%. This high heterogeneity is not uncommon and may be attributed to the fact that the control diets differed in each included study, and also, some studies had uneven sample sizes. The heterogeneity should be considered when interpreting the results; however, in this systematic review, the heterogeneity of the included studies had no influence on clinical decision-making since the superiority of both low-carbohydrate and KDs compared with the control diets was well established. The study also included studies that independently compared ketogenic or low-carbohydrate diets to other diets due to the lack of studies that directly compare ketogenic to low-carbohydrate diets among patients with T2DM. This may result in biased results since the control diets differed in each included study. The study also included studies published only in English, which may lead to the omission of important information that may have been retrieved from the studies published in other languages.

## Conclusions

This systematic review suggests that both ketogenic and low-carbohydrate diets produce significant differences in HbA1c levels, which is a measure of glycemic control. However, comparing the effects of ketogenic and low-carbohydrate diets on glycemic control, it is evident that KDs had a significant reduction in HbA1c values compared to low-carbohydrate diets. This difference can be attributed to the fact that glycemic control mostly depends on the degree of carbohydrate restrictions. The results also show that a significant weight loss is observed in patients subjected to KDs compared to those subjected to low-carbohydrate diets. This increased weight loss observed in T2DM patients subjected to a KD can be associated with a significant decrease in HbA1c. No significant difference in BMI values was observed in either ketogenic or low-carbohydrate diets. These results show that the KD can be considered an effective dietary therapy for patients with T2DM. However, for patients with T2DM, it is necessary to balance the potential increase in cardiovascular risk, which results from an unfavourable blood lipid profile observed with a KD derived from the weight loss and improved glycemic control.
